# Characterization of HSP70 and HSP90 Gene Family in *Takifugu fasciatus* and Their Expression Profiles on Biotic and Abiotic Stresses Response

**DOI:** 10.3390/genes15111445

**Published:** 2024-11-08

**Authors:** Wenwen Zhang, Ziang Qian, Jie Ji, Tao Wang, Shaowu Yin, Kai Zhang

**Affiliations:** 1 Jiangsu Province Engineering Research Center for Aquatic Animals Breeding and Green Efficient Aquacultural Technology, College of Marine Science and Engineering, Nanjing Normal University, Nanjing 210023, China; zhangwenwen1107@163.com (W.Z.); ziangqian@163.com (Z.Q.); jie.ji@njnu.edu.cn (J.J.); 08318@njnu.edu.cn (T.W.); yinshaowu@163.com (S.Y.); 2 State Key Laboratory of Mariculture Breeding, College of Ocean and Earth Sciences, Xiamen University, Xiamen 361102, China; 3 Co-Innovation Center for Marine Bio-Industry Technology, Lianyungang 222005, China

**Keywords:** heat shock protein, *Takifugu fasciatus*, biotic and abiotic stresses, innate immunity, expression profile

## Abstract

Background: Heat shock proteins (HSPs) play crucial roles in response to temperature changes and biotic stresses. However, the HSP gene family in the pufferfish (*Takifugu fasciatus*) herring has not been comprehensively investigated. Methods and Results: This study presents a systematic analysis of the HSP70 and HSP90 gene families in *T. fasciatus*, focusing on gene characterization, conserved structural domains, molecular evolutionary history, and expression patterns of the HSP gene family under stress conditions. The findings reveal that 16 HSP genes are evolutionarily conserved, while *hspa4* and *hsp90aa* appear specific to teleost fish. *HSP* genes exhibit widespread expression across 11 examined tissues, with most demonstrating high expression levels in the heart, brain, and liver. Furthermore, *T. fasciatus* was subjected to cryogenic and biotic stresses, revealing distinct expression patterns of *HSPs* under various stress conditions. The response of *HSPs* to cold stress and *Aeromonas hydrophila* infection was sustained. In contrast, gene expression of *HSPs* significantly changed only in the pre-infection period following *Ichthyophthirius multifiliis* infection, gradually returning to normal levels in the later stages. Conclusions: These experimental results provide a foundation for further in-depth investigations into the characteristics and functions of HSPs in *T. fasciatus*.

## 1. Introduction

Heat shock proteins (HSPs) are molecular chaperones widely found in plants, animals, and microorganisms [1]. Under normal conditions, HSPs play a pivotal role in maintaining the stability of the cellular environment by assisting in protein folding and assembly. They prevent the aggregation of denatured proteins, promote the degradation of misfolded or aggregated proteins, and assist in protein refolding, thereby mitigating the deleterious effects of environmental stresses and enhancing cellular stress tolerance [2,3]. The expression of heat shock proteins (HSPs) is regulated by heat shock transcription factors (HSFs) that act by binding to heat shock elements (HSEs) located in the promoter region of the HSP gene [4]. HSPs were initially discovered in the salivary glands of *Drosophila melanogaster* subjected to heat stress. Since this seminal finding, extensive research has been conducted on HSPs in both prokaryotic and eukaryotic organisms [5]. It is now well established that intracellular HSP levels are modulated not only in response to temperature stress but also in response to various abiotic [6], biotic [7], and chemical stressors [8].

HSPs are highly conserved in both eukaryotes and prokaryotes and are generally classified according to the molecular weight of the proteins [9]. The HSP70 family, which is crucial for protein synthesis, transport, and storage, comprises the majority of known gene sequences and is the most important HSP family member in organisms [10]. Similarly, HSP90 is a key chaperone protein that stabilizes proteins involved in the malignant transformation of cells, maintaining the proliferative potential of cells and preventing apoptosis [11]. The whole genome sequences of various organisms have revealed numerous gene-encoding proteins within the HSP gene families. For instance, *Liriodendron chinense* possesses 23 hsp70 family members and 7 hsp90 family members [12], while *Aedes aegypti* has 17 hsp70 family members and 4 hsp90 family members [3], and *Trachinotus ovatus* contains 13 hsp70 family members and 3 hsp90 family members [13].

As ectothermic organisms, fish frequently encounter a range of environmental factors (especially temperature) during the growth and reproduction phases, making fish an excellent model for studying HSP expression and function [14]. Although temperature stimulation induces changes in HSP expression, current research primarily focuses on the patterns and mechanisms of HSP expression under high-temperature stress [15]. There is a lack of research on HSP expression under cold stress conditions. Moreover, in aquaculture systems, fish are susceptible to a wide range of pathogens, leading to significant economic losses in the fishing industry. Two common pathogens affecting cultured fish are *A. hydrophila* and *I. multifiliis* [16]. *A. hydrophila*, a Gram-negative bacillus, is a major pathogen causing hemorrhagic septicemia in fish and is widely distributed in fresh and marine waters, capable of infecting a diverse range of aquatic animals [17]. *I. multifiliis* is a highly pathogenic parasite of cultured fish, characterized by its ability to penetrate tissues, causing destruction and increasing vulnerability to other pathogens, ultimately leading to decreased immunity in infected fish [18]. Currently, there is a lack of clarity regarding the mechanisms by which the fish HSP gene family responds to these two pathogens.

*T. fasciatus* is an important warm-water migratory economic fish with high nutritional value and popularity in China. With the expansion of the intensive breeding scale of *T. fasciatus*, bacterial [19], parasitic [20], and temperature-related [21] diseases have become increasingly problematic. To date, there have been no reports on the identification and systematic analyses of the HSP gene family in *T. fasciatus* nor on their response to cold stress and pathogen infection. In this study, the HSP70 and HSP90 gene families (hereinafter referred to as HSPs) were comprehensively analyzed in *T. fasciatus*, including their identification, biological characterization, phylogeny, chromosomal locations, selective pressure, and expression patterns under infection by two pathogens and cold stress. The findings provide a valuable resource for further understanding the regulatory mechanisms of the HSP gene family in *T. fasciatus* under low-temperature, bacterial, and parasitic stresses.

## 2. Materials and Methods

### 2.1. Identification of HSP70 and HSP90 Gene Families

HSP70 and HSP90 genes and protein sequences of Japanese pufferfish (*Takifugu rubripes*) were obtained from the National Center for Biotechnology Information (NCBI) database (http://www.ncbi.nlm.nih.gov, accessed on 5 November 2024) [22]. After removing the redundant sequences, the target genes were screened using BLASTN and TBLASTN through repeated searches in the *T. fasciatus* genome database, applying a threshold of an e-value < 1 × 10^−6^. The protein sequences of the screened genes were submitted to the Simple Modular Architecture Research Tool (SMART) (http://smart.embl.de, accessed on 5 November 2024) to confirm the integrity of conserved structural domains [23]. Finally, the genes of the HSP70 and HSP90 gene families were named according to their homology with the respective gene families in *T. rubripes*.

### 2.2. Bioinformatic Analysis of TfHSP70 and HSP90 Gene Families

The complete coding sequence was of HSPs predicted using ORF Finder (https://www.ncbi.nlm.nih.gov/orffinder/, accessed on 5 November 2024) [24]. Physicochemical properties of HSP70 and HSP90 proteins were predicted using the online software ExPASy ProtParam (https://web.expasy.org/protparam/, accessed on 5 November 2024) [25]. The location information of HSP70 and HSP90 genes was obtained from the annotation file of the *T. fasciatus* genome, drawn from the chromosome location map by MapGene2Chrom web v2 (http://mg2c.iask.in/mg2c_v2.0/, accessed on 5 November 2024) [26]. The conserved motifs of the full-length HSP70 and HSP90 family proteins were analyzed using the online MEME tool (http://meme-suite.org/tools/meme, accessed on 5 November 2024), with the maximum motif search value set at 10 [27]. Furthermore, WoLF PSORT (https://www.genscript.com/psort.html, accessed on 5 November 2024) was used for subcellular localization prediction [28].

### 2.3. Phylogenetic Tree Construction

To construct the phylogenetic tree, information on the HSPs of other species was utilized. All protein sequences were obtained through BLAST searches downloaded from NCBI (http://www.ncbi.nlm.nih.gov/BLAST/, accessed on 5 November 2024), with the accession numbers listed in Table S2. The MUSCLE program was employed to align all HSPs sequences, and IQ-tree 1.6.12 software was used to construct the phylogenetic trees. The tree-building alternative model for the HSP70 family was JTT+I+G4, while LG+G4+F was used for the HSP90 family, both with a Bootstrap count of 1000.

### 2.4. Molecular Evolution Analysis

To comprehensively analyze the selective pressures on *T. fasciatus* HSPs across fish populations, we used a fish phylogenetic tree and selected representative fish HSPs from a broad range of taxa for inclusion in this analysis. To obtain information about natural selection pressures, we generated non-synonymous (dN) versus synonymous substitution (dS) rates for different HSPs. All amino acid sequences were obtained from the GeneBank database, and the accession numbers are listed in Table S3. Selection pressure on each codon site of HSPs was predicted using the single-likelihood ancestor counting (SLAC) method, which is based on maximum likelihood estimation of non-synonymous and synonymous substitution rates [29]. These analyses were performed using the Datamonkey web server (http://www.datamonkey.org/, accessed on 5 November 2024) [30], allowing for the assessment of selection pressure on different regions of the two gene families and providing insight into the molecular evolution of this gene family.

### 2.5. Experimental Animals

Pufferfish (26.24 ± 2.18 g, mean body weight) were obtained from Zhongyang Group Co., Ltd. (Nantong, China) and immediately transported to the laboratory for a 7-day acclimatization period. The fish were kept in biofiltration recirculating water systems containing 100 L of fresh water (equipped with cooling and heating functions; temperature, 25.0 ± 0.5 °C; pH 7.5; dissolved oxygen 7.0 mg/L; salinity 0.2 ± 0.1 ppt). During this period, the fish were fed commercial powder diets (Grobest, Foshan, China) twice daily (08:00 and 18:00) at 5% of body weight and fasted for 24 h before the experiments.

To clone the full-length complementary DNA (cDNA) and analyze the tissue distribution expression profile of HSP70 and HSP90 genes, nine fish were sampled for 11 organs/tissues (liver, gill, gonad, spleen, brain, intestine, kidney, eye, skin, heart and muscle) in a consistent manner.

### 2.6. Cold Stress

Pufferfish acclimated to a temperature of 25–32 °C, and died at 11 °C for significant frostbit. Therefore, we set the treatment groups at 19 °C and 13 °C and randomly divided 180 healthy pufferfish into control and treatment groups with three replicates in each group. The water temperature of the control group was maintained at 25.0 ± 1.0 °C throughout the experimental period. Following the procedures from our previous studies [31], the water temperature of the two cold stress groups was decreased from 25 °C to 19 °C at a constant rate of 0.85 °C/1 h. One group was maintained at 19 °C until the end of the exposure, while the other group was kept at 19 °C for 12 h before the temperature was further decreased to the preset 13 °C at the same rate. In the treatment groups, the formal experiment commenced once the temperature reached the target values, and this time point was recorded as 0 h. After anesthesia in 0.05% MS-222 (Sigma, St. Louis, MO, USA), nine fish from each group were dissected at 0, 6, 24, and 96 h of cold exposure. Livers and intestines were quickly sampled, pooled in three biological replicates, frozen in liquid nitrogen, and preserved at −80 °C for further HSP gene pattern analysis.

### 2.7. A. hydrophila Infection

An infection experiment using *A. hydrophila* was conducted on pufferfish. Briefly, the *A. hydrophila* strain, isolated from farmed pufferfish in China’s Jiangsu province, was purified, cultured, and stored at −80 °C with glycerol. Healthy, uniform-sized pufferfish were randomly divided into two groups. A concentration gradient pre-test yielded the lethal dose 50 of approximately 1 × 10^9^ CFU/mL for *A. hydrophila* on *T. fasciatus*, so the cultured *A. hydrophila* concentration was adjusted to this concentration using PBS buffer. The treatment group received 0.1 mL of the bacterial solution via intraperitoneal injection in the tail, while the control group was injected with 0.1 mL of PBS buffer. Three pufferfish from each group were dissected and liver tissue samples were dissected at 6, 12, and 24 h post injections.

### 2.8. I. multifiliis Infection

After excluding the possibility of exogenous factor stimulation, nine healthy pufferfish in the same tank were infected with *I. multifiliis* under laboratory conditions using a prepared *I. multifiliis* solution (1000 theronts/L, 100 L). During the infection process, the internal biofilter was closed, and water exchange was halted. A microscopic examination of the gills and skin of pufferfish, conducted following the observation of white spots, confirmed the presence of *I. multifiliis*. On the third and tenth day post-infection, three pufferfish were randomly selected for the experimental group, while three healthy pufferfish were randomly chosen for the control group, and the liver tissues of the pufferfish were collected.

### 2.9. Isolation of RNA, Synthesis of cDNA and Quantitative Real-Time PCR (qRT-PCR)

Total RNA was extracted from 11 untreated tissues, cold treatment tissues, *A. hydrophila* and *I. multifiliis* infection tissues and reverse transcribed to cDNA according to the previously reported method [32]. qRT-PCR was performed on a LightCycler96 system (Roche, Basel, Switzerland) following the manufacturer’s protocol. The specific primer pairs of *T. fasciatus* HSPs were designed according to the known sequences found in the National Center for Biotechnology Information (NCBI) database (Table S1). The *β-actin* gene was used as an internal reference gene. The relative expressions of genes were calculated using the 2^−ΔΔCt^ method [33].

### 2.10. Statistical Analysis

All data were expressed as mean ± standard deviation (SD). Statistical analyses were performed using SPSS 25.0 software (Statistical Package for the Social Sciences, Chicago, IL, USA). The gene expression data were examined for homogeneity and the normality of variance using Levene and Kolmogorov–Smirnov (K-S) tests, respectively. When the data met the assumptions of homogeneity and normality (*p* > 0.05), one-way analysis of variance (ANOVA) and Fisher’s Least Significant Difference (LSD) multiple comparison tests were employed. If the data did not meet these assumptions, the Kruskal–Wallis (K-W) test was used for the analysis. Graphics were generated using GraphPad Prism software 8.0 (GraphPad, Inc., La Jolla, CA, USA).

## 3. Results

### 3.1. Identification and Characteristic Analysis of T. fasciatus HSP70 and HSP90

A search of the *T. fasciatus* genome database in our laboratory using BLAST identified 16 HSP family sequences, including 11 HSP70 and 5 HSP90 sequences. Table 1 lists the relevant features of these genes, such as the number of amino acids, molecular weight, isoelectric point, transmembrane regions, signal peptides, location of gene family structural domains, and NCBI accession numbers. The sequence lengths ranged from 441 to 853 amino acids, with predicted molecular weights of 48.29–95.98 kDa and theoretical isoelectric points of 4.73–8.57. The molecular features were more similar among the five HSP90 gene family members, and the computed pI values indicated the acidic nature of the proteins. Transmembrane and signal peptide regions were essentially absent in the HSP gene family members. Subcellular localization prediction analysis revealed that HSPs were primarily present in the cytosol, with a few found in the endoplasmic reticulum and mitochondrion.

### 3.2. Phylogenetic Analysis

**Figure 1:**
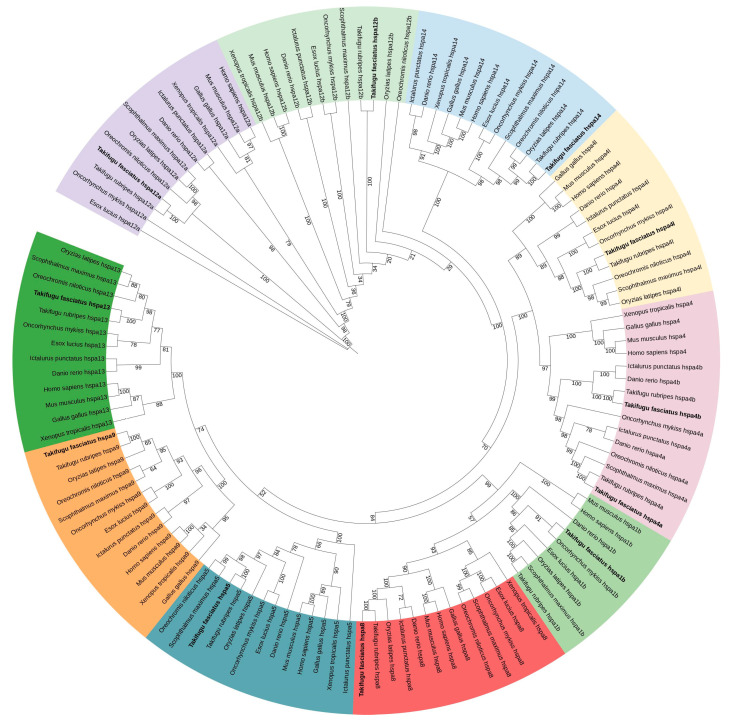
Phylogenetic analysis of *T. fasciatus* HSP70 proteins. Different subfamilies are distinguished by different colors. Numbers represent bootstrap percentages.

**Figure 2:**
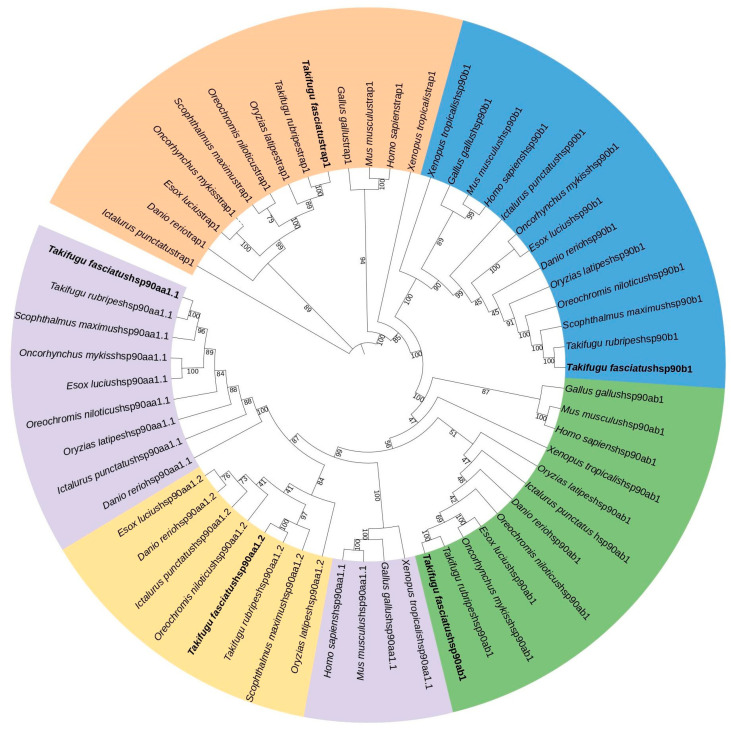
Phylogenetic analysis of *T. fasciatus* HSP90 proteins. Different subfamilies are distinguished by different colors. Numbers represent bootstrap percentages.

To ascertain the genetic evolutionary relationships among all HSPs, we constructed a phylogenetic tree using HSP protein sequences from *T. rubripes*, *Scophthalmus maximus*, *Oreochromis niloticus*, *Ictalurus punctatus*, *Danio rerio*, *Oncorhynchus mykiss*, *Esox lucius*, and *Oryzias latipes* (Figure 1 and Figure 2). The phylogenetic trees demonstrate that the HSPs are highly conserved in teleost and that *T. fasciatus* exhibits the closest evolutionary relationship to *T. rubripes*, a member of the same genus. Notably, teleost fish possess both hspa4a and hspa4b subtypes within the HSP70 gene family, whereas higher organisms, such as mammals, reptiles, and birds, lack the hspa4 subtype. Similarly, within the HSP90 gene family, teleost fish contain both hsp90aa1.1 and hsp90aa1.2 isoforms, while higher organisms, including mammals, reptiles, and birds, possess only hsp90aa1.1 family members. Intriguingly, the hsp90aa1.1 gene in higher organisms exhibits a closer evolutionary relationship to the hsp90aa1.2 gene in teleost fish.

### 3.3. Conserved Motifs, Structural Domains, and Chromosomal Localization of Members of the HSP70 and HSP90 Gene Families

**Figure 3:**
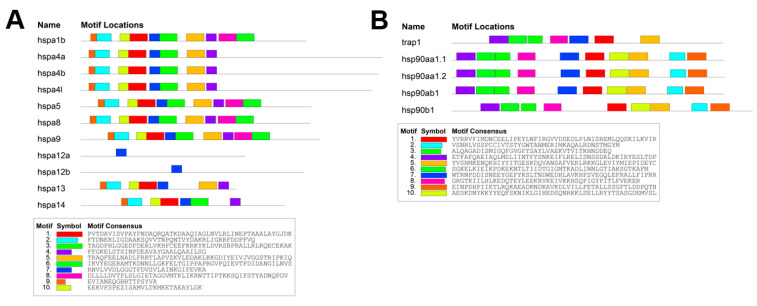
Conserved motif and gene structure of HSPs in *T. fasciatus*. Different colors indicate different conserved motifs. (**A**): Conserved motifs of the HSP70 gene family. (**B**): Conserved motifs of the HSP90 gene family.

To further explore the structural features of the HSP gene family of *T. fasciatus*, the conserved 10 putative motifs of the HSP genes of *T. fasciatus* were analyzed and visualized using the MEME online website (Figure 3). The results indicated that the length of these conserved motifs ranged from 17 to 50 amino acids. All motifs were absent except in hspa1b, hspa5, hspa8, and hspa9, which contained motifs 1–10. Notably, hspa12a and hspa12b were missing most of the conserved motifs, with only motif 7 being retained (Figure 3A). In contrast, the number of motifs in the HSP90 gene family varied from 7 to 10, with hsp90b1 lacking motif 7 and trap1 missing motifs 2, 9, and 10 (Figure 3B).

**Figure 4:**
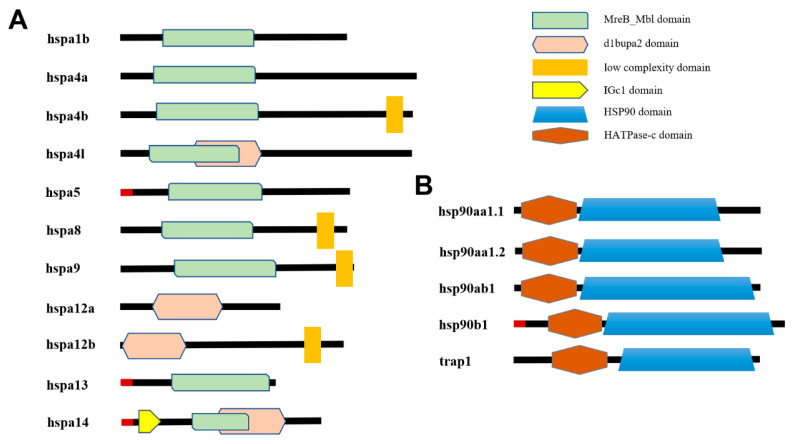
The distribution map of *T. fasciatus* (**A**) HSP70 and (**B**) HSP90 domain. The red part at the top indicates the signal peptide region.

The structures of HSP70 and HSP90 were further characterized (Figure 4). For specific locations of HSP70 domains not represented in the figure, refer to Table 2. The majority of HSP70 gene family members contain similar MreB_Mbl conserved structural domains, suggesting potential functional similarities. Additionally, some members possess low complexity regions (LCRs), which are composed of amino acid sequences containing single amino acid repeats or short amino acid patterns [34]. However, hspa12a and hspa12b contain only the d1bupa2 structural domain, which is also present in hspa4l and hspa14 (Figure 4A). As illustrated in Figure 4B, evolutionarily conserved domains were identified in five HSP90 proteins, with all HSP90 proteins sharing an HATPase-c domain. In summary, the HSP70s and HSP90 in *T. fasciatus* exhibit a high degree of conservation.

**Figure 5:**
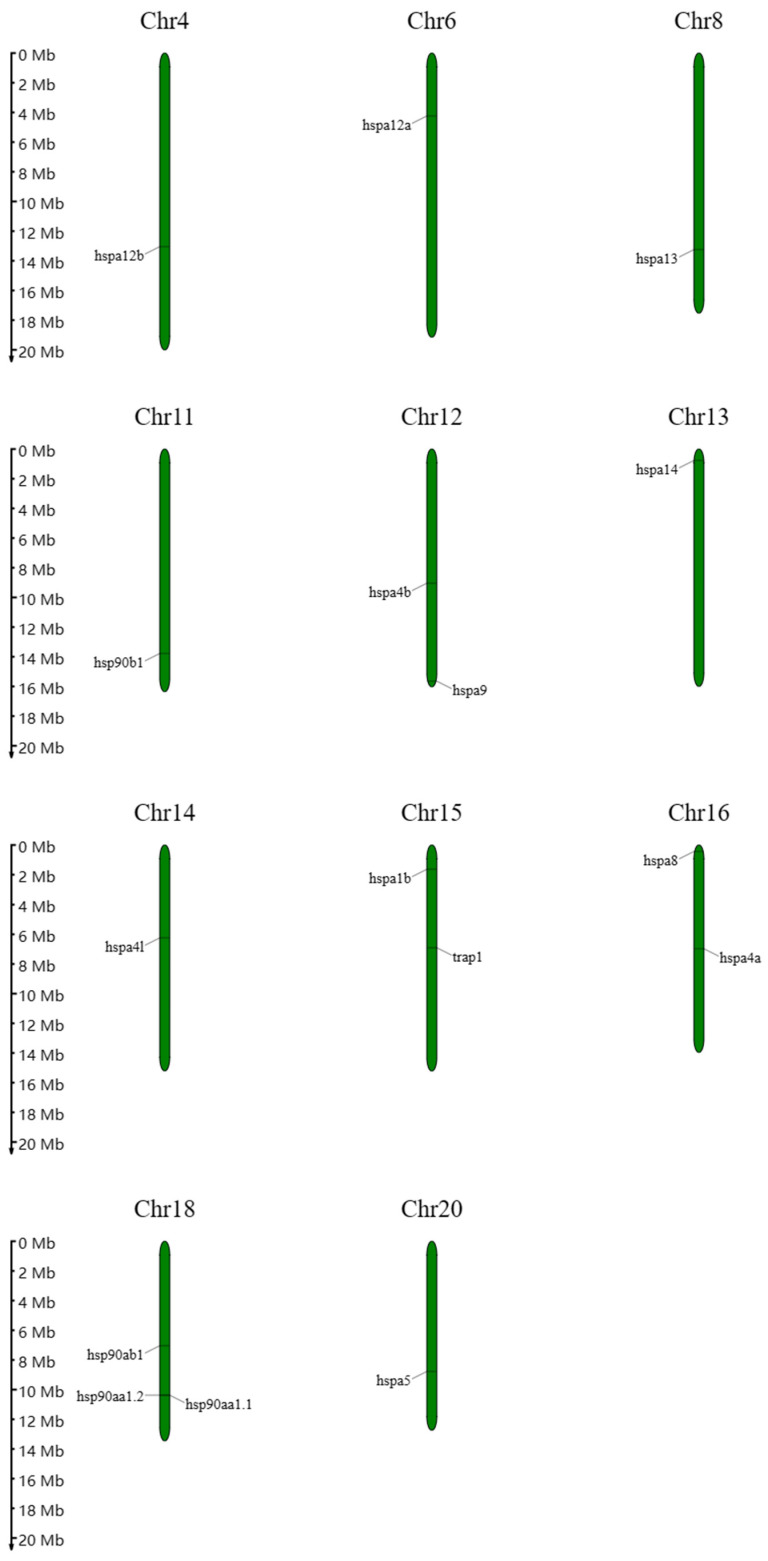
Chromosomal location HSP70 and HSP90 gene families in *T. fasciatus* chromosomes.

The localization of HSPs is depicted in Figure 5. The HSP90 genes are primarily situated on chromosomes 11, 15, and 18. In contrast, the HSP70 genes exhibit a nonuniform distribution across the nine chromosome scaffolds of the *T. fasciatus* scaffold, with locations on chromosomes 4, 6, 8, 12, 13, 14, 15, 16, and 20.

### 3.4. Molecular Evolution Analysis of HSP70 and HSP90 Gene Family Members

To better understand the high interspecific evolutionary history of the HSPs, we calculated the ratios of non-synonymous to synonymous substitutions (dN/dS) using the CDS of HSPs across various teleost fish species. As shown in Table 2, the dN/dS ratios for the HSP70s range from 0.0576 to 0.165, while the dN/dS ratios for the HSP90s vary from 0.0462 to 0.100, indicating that both HSP70s and HSP90s are under purifying selection and likewise suggesting that these genes are subjected to negative selective pressures and are relatively conserved. Furthermore, we identified a range of negatively selected sites in HSPs (74–257), suggesting that these sites lack mutant alleles, are evolutionarily conserved, and may have important functions. Additionally, no positively selected sites were found in any of the HSP70s or HSP90s.

### 3.5. Tissue Expression Profiling of HSP70 and HSP90 Gene Family Members

**Figure 6:**
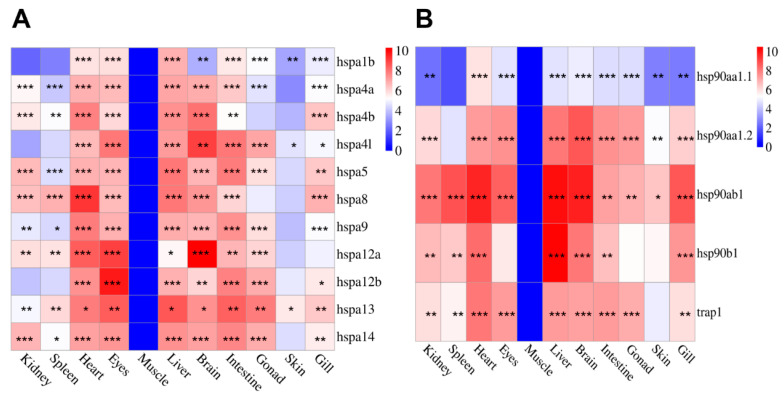
Heatmap of *HSP70* and *HSP90* genes expression in different tissues at 25 °C. Each block in the heatmap represents the expression level of *HSP70* and *HSP90* genes, which are normalized to log^10^. Different asterisks “*” indicate statistical significance (* *p* < 0.05, ** *p* < 0.01, and *** *p* < 0.001). (**A**): Expression pattern of *HSP70* in different tissues. (**B**): Expression pattern of *HSP90* in different tissues.

The tissue distribution of *HSP70* and *HSP90* gene transcription at 25 °C was detected using qRT-PCR. The expression patterns of *HSP* genes in unchallenged tissues were visualized using heat maps. The results showed that *HSP70* and *HSP90* gene families were present in all tissues. As presented in Figure 6A, the *HSP70* signals are detected in all tested tissues, with the strongest expression in the eyes, liver, brain, and heart. In contrast, there was low expression in muscle and skin. Similarly, *HSP90*s had the lowest mRNA expression levels in muscle tissues and were predominantly expressed in the liver, heart, and brain (Figure 6B). However, the expression levels among different members differed significantly; for example, the *hspa1b* gene was most highly expressed in the liver, but the *hspa12a* gene was most highly expressed in the brain with a relatively low expression in liver tissue.

### 3.6. Expression Profiles of HSP70s and HSP90s in Cold Stress

**Figure 7:**
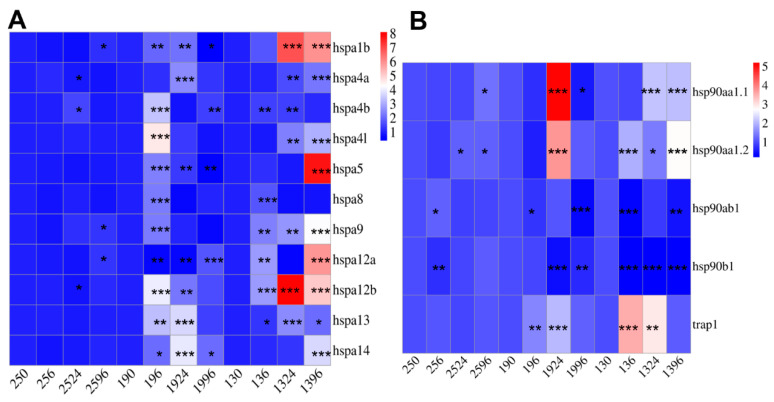
Heatmap of *HSP70* and *HSP90* expression in the liver after temperature stress. The numbers below graphics are structured in two parts with the first two characters represent temperature and the last two indicating exposure time. Different asterisks “*” indicate statistical significance (* *p* < 0.05, ** *p* < 0.01, and *** *p* < 0.001). (**A**): Expression of *HSP70*s in cold stress. (**B**): Expression of *HSP90*s in cold stress.

The expression levels of *HSP70*s and *HSP90*s under cold stress were analyzed by qRT PCR, and the data from this assay were subsequently used to generate a heat map for presentation (Figure 7). In the 13 °C group, the expression levels of *HSP70*s exhibited a distinct time of exposure-dependent pattern. Compared with 0 h of cold stress, the mRNA of the *HSP70* gene showed a gradual increase, with all isoforms peaking at 13 °C 96 h (Figure 7A). Conversely, *hsp90b1* and *hsp90ab1* showed a significant decrease in expression following cold stress. *hsp90aa1.1* and *hsp90aa1.2* demonstrated a similar expression pattern, peaking at 19 °C for 24 h, while *trap1* exhibited an increasing followed by a decreasing trend, with a significant upward trend at the onset of the cold stress (Figure 7B).

### 3.7. Expression Patterns of HSP70 and HSP90 Gene Family Members in Response to A. hydrophila

**Figure 8:**
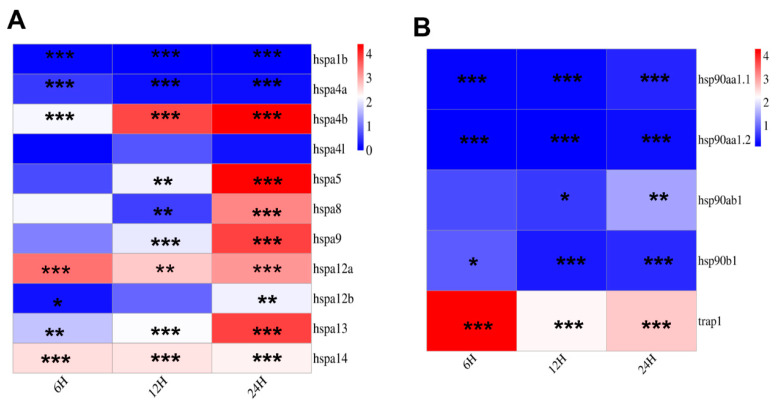
Heatmap of *HSP70* and *HSP90* gene expression in the liver after *A. hydrophila* infection. Different asterisks “*” indicate statistical significance (* *p* < 0.05, ** *p* < 0.01, and *** *p* < 0.001). (**A**): Expression of *HSP70*s after *A. hydrophila* infection. (**B**): Expression of *HSP90*s after *A. hydrophila* infection.

Differential expression analysis revealed significant alterations in the expression of all HSPs, with the exception of the *hspa4l* gene, following *A. hydrophila* infection. The majority of *HSP70*s exhibited a time-dependent significant upregulation in expression, while the *hspa1b* and *hspa4a* genes demonstrated reduced expression post-*A. hydrophila* infection (Figure 8A). Conversely, the expression of *HSP90*s decreased as the duration of *A. hydrophila* infection increased, reaching a maximum at 24 h post infection. However, *trap1* displayed an opposing trend, showing an upward trajectory (Figure 8B).

### 3.8. Expression of HSP70 and HSP90 Gene Family Members After I. multifiliis Infection

**Figure 9:**
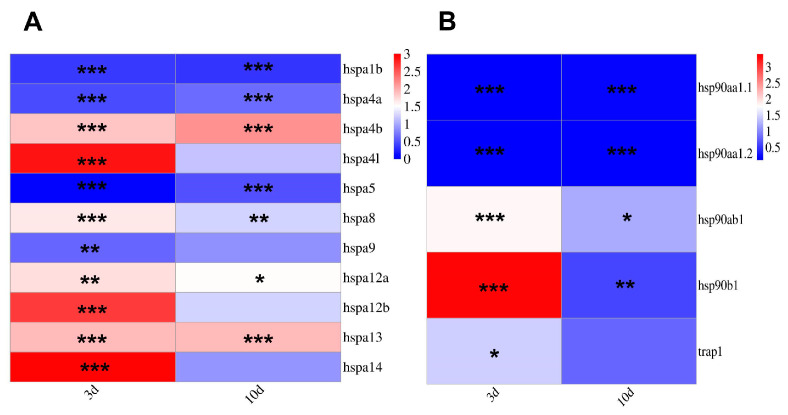
Heatmap of *HSP70* and *HSP90* gene family member expression in the liver after *I. multifiliis* infection. Different asterisks “*” indicate statistical significance (* *p* < 0.05, ** *p* < 0.01, and *** *p* < 0.001). (**A**): Expression of *HSP70*s after *I. multifiliis* infection. (**B**): Expression of *HSP90*s after *I. multifiliis* infection.

The qRT-PCR was employed to examine the expression pattern of *HSP*s following the parasitism of *I. multifiliis* on *T. fasciatus*. The results demonstrated that the expression of over half of the *HSP70* gene family members was significantly upregulated during the early stage of *I. multifiliis* infection and exhibited a declining trend after 10 days of infection. The expression levels of *hspa4l*, *hspa9*, *hspa12b*, and *hspa14* returned to baseline levels (Figure 9A). Among the *HSP90* gene family members, with the exception of *hsp90ab1* and *hsp90b1*, which displayed an increasing trend at the onset of infection, the remaining members showed a significant decreasing trend following *I. multifiliis* infection (Figure 9B).

## 4. Discussion

*T. fasciatus* is one of the most important economic species in China, and the current aquaculture production in China is showing an upward trend [35]. Previous studies have shown that *A. hydrophila* infections [36], parasitism by *I. multifiliis* [37], and temperature variations [38] cause significant economic losses to farmed fish. HSPs are widely used as biomarkers for a vast array of stressors in different organisms [39]. The liver is susceptible to impaired energy metabolism by low temperatures and is extremely sensitive to the external environment, making it a good model for studying the expression patterns of HSPs [40]. However, a systematic identification of the HSP gene family members in *T. fasciatus* has not been reported.

The present study identified 11 HSP70 genes (*hspa1b*, *hspa4a*, *hspa4b*, *hspa4l*, *hspa5*, *hspa8*, *hspa9*, *hspa12a*, *hspa12b*, *hspa13*, and *hspa14*) and 5 HSP90 genes (hsp90aa1.1, hsp90aa1.2, hsp90ab1, hsp90b1, and trap1) to further elucidate their roles in the stress response process of fish. Most HSP genes function in the cytoplasm and members of the same family exhibit similar molecular features. A comparison of the identified genes revealed that the number of HSP genes in *T. fasciatus* and other fish species is higher than in other organisms. The variation in HSP gene repertoire across different organisms may be attributed to the acquisition of HSP members in the genomes of different taxa throughout the process of species evolution and divergence.

These genes also possess HSP signature domains. Additionally, the MreB_Mbl domain was identified in *Tf*HSP70s, consistent with findings from a previous study by Dayrit [41]. Hsp70, a chaperone, belongs to a larger superfamily that includes actin, which may have originated from a prokaryotic precursor protein associated with MreB/Mbl [42]. This structural domain, therefore, indicates an evolutionary connection to HSP70. Notably, the analysis also revealed that HSP90s contain the HATPases-c domain, aligning with their function as molecular chaperones [43]. Evolutionary tree analysis, demonstrating a high similarity between HSP70s and HSP90s in *T. fasciatus* and those in teleost fish, further supports the evolutionary conservation of these proteins in fish.

The dN/dS ratio, defined as the ratio of non-synonymous to synonymous substitution rates, is widely used to assess selection pressures acting on protein-coding genes [44]. In this study, the dN/dS ratio of all HSP genes was found to be less than 1, indicating that natural selection is acting to suppress protein changes. This finding suggests that the genes are likely subject to purifying selection, characterized by the absence of non-synonymous nucleotide changes at the codon level. The results demonstrate that the HSP70s and HSP90s among the analyzed teleost species are evolutionarily constrained and functionally conserved.

The expression pattern of *HSP*s across various tissues in *T. fasciatus* revealed ubiquitous expression, albeit with significant differences in levels among different members. Most members exhibited the highest expression in liver tissue. The liver, a central organ for maintaining systemic energetic homeostasis [45] and an important component of the innate immune system [46], is highly sensitive to environmental changes and plays a crucial role in immune defense in scleractinian fish. Thus, the elevated expression of *HSP70* and *HSP90* genes in liver tissue suggests their potential involvement in metabolic processes. Following acute cold stress, most *HSP* genes in *T. fasciatus* hepatocytes demonstrated an upward trend, implying the role of *HSP70* and *HSP90* in coping with such stress. Convincing evidence supports the essentiality of the HSP70 gene in maintaining homeostasis in fish liver under stress conditions during cold acclimatization [47]. *HSP90*s contribute to the maintenance of cellular and organelle structures [48] and may protect cellular compartments from temperature-induced damage [43]. An investigation into the expression patterns of five *HSP70* gene family members (*hspa1a*, *hspa4*, *hspa8*, *hspa9*, *and hspa14*) in breast cancer cell lines following low-temperature treatment revealed increased expression in all members except *hspa4* [49], aligning with the findings of the present study.

HSP activates and modulates the cellular immune response and mitigates the effects of pathological stress on organisms [5]. HSP70 is reported to be one of the most thermally inducible and protective proteins involved in the immune response of teleost fish, from a non-specific to an adaptive immune response [50]. In *Labeo rohita*, HSP gene regulation was greater in the liver than in the spleen and kidney tissues, with *HSP70* increasing and *HSP90* briefly increasing at the beginning of infection (6 h) before decreasing [51]. However, the complexities of fish bacterial pathogens require a consideration of extracellular product (ECP) production. *Sparus sarba* did not alter *hsp90* expression after infection with *Vibrio alginolyticus*, while ECP significantly reduced hepatic *hsp90* in late acute infection, and *hsp70* levels rapidly and drastically increased with both live *V. alginolyticus* and ECP [52]. Liver and kidney *HSP90* remained unchanged during the natural time course of infection after the natural exposure of silver seabream to *V. alginolyticus* [53]. Hepatocyte necrosis in *Anguilla rostrata* infected with *A. hydrophila* and transcriptome analysis reveals differential genes involved in immunity including members of the *HSP90* gene family [54]. In the present study, most *HSP70* family members were significantly up-regulated in response to *A. hydrophila* infection, suggesting their involvement in resistance to pathogenic bacteria. However, most *HSP90* family members were significantly down-regulated after infection, possibly due to higher concentrations of factors in the ECP fraction that lead to reduced *HSP90* levels, either by directly interfering with liver function or through more complex signaling pathways [5]. Notably, *hsp90ab1* showed an upward trend in the later stages of infection, a trend also found in *Solea senegalensis* infected with *Photobacterium damselae* [55], suggesting that expression changes in *HSP90* gene family members may manifest only in the later stages of infection.

White spot disease, caused by *I. multifiliis*, is one of the most severe diseases affecting fish [56]. HSPs have been demonstrated to play an important role in the invasion of *I. multifiliis*. These proteins may serve as valuable markers of the pathological condition in *T. fasciatus*, as their expression is upregulated during *I. multifiliis* infection [46]. Most of the *HSP*s were found in *O. mykiss* liver showing a compassionate response to *I. multifiliis* infection, which is consistent with the results of the present experiments [57]. In addition, the present study found that most *HSP*s exhibited a significant increase in expression during the early stage of infection, followed by a return to normal levels or a significant decrease in the late stage. This suggests that *HSP* genes are involved in resistance to pathogenic bacteria and may play a critical role in the early stages of infection. Another study demonstrated that a subset of the *HSP70* family members, among others, were highly expressed in both the *I. multifiliis* infection group and the temperature stress group [57]. These findings simultaneously demonstrate the reliability of the experimental results in this paper and support the sensitivity and potential use of specific *HSP70* family members as biomarkers of stress and disease. Furthermore, the present study found that *hspa12a* and *hspa12b* were upregulated under three different stress conditions, indicating that the *hspa12* subfamily plays a significant role in the stress response during abiotic stress and/or biodefense. This finding has also been demonstrated in scallops [58]. The *hspa12* subfamily is generally recognized for its protective function in response to a variety of stressors [59].

## 5. Conclusions

In this study, 16 HSP genes belonging to two gene families were identified and screened for *T. fasciatus*. The results demonstrated that members of the HSPs were highly conserved in teleost fish. The majority of the HSP genes responded during the pre-cold stress period. Furthermore, the expression profiles of *HSP*s in response to bacterial infection and parasite invasion differed. This study suggests the significant roles of HSP70 and HSP90 in teleost immunity under various stressors. Further research is necessary to explore their potential functional mechanisms in teleost host immune activities.

## Figures and Tables

**Table 1:** Basic characteristics of HSP70 and HSP90 gene families.

Gene Family	Gene Name	Number of Amino Acids	Molecular Weight (Da)	Isoelectric Point	Subcellular Localization	Transmembrane Region	Signal Peptide	HSP70/HSP90 Domain Location (aa)	NCBI Accession Number
HSP70 family	*hspa1b*	639	70,183.16	5.23	Cytosol	0	0	8–614	PQ469192
	*hspa4a*	853	95,982.99	5.25	Cytosol	0	0	3–593	PQ469188
	*hspa4b*	844	94,489.05	5.00	Cytosol	0	0	3–609	PQ469189
	*hspa4l*	825	92,634.99	5.39	Cytosol	0	0	3–688	PQ469187
	*hspa5*	653	72,248.71	5.02	Endoplasmic reticulum	0	17–18	29–634	PQ469193
	*hspa8*	650	71,370.38	5.26	Cytosol	0	0	6–612	PQ469184
	*hspa9*	678	73,808.51	5.54	Mitochondrion	0	0	57–655	PQ469185
	*hspa12a*	465	52,054.89	8.57	Cytosol	0	0	95–343	PQ469186
	*hspa12b*	633	70,569.25	8.50	Cytosol	0	0	94–531	PQ469191
	*hspa13*	441	48,295.41	6.39	Mitochondrion	0	24–25	33–430	PQ469194
	*hspa14*	578	63,341.75	7.34	Peroxisome	0	19–20	398–578	PQ469190
HSP90 family	*hsp90aa1.1*	724	83,283.81	5.04	Cytosol	0	0	192–714	PQ469195
	*hsp90aa1.2*	727	83,904.85	4.90	Cytosol	0	0	192–716	PQ469196
	*hsp90ab1*	723	83,119.97	4.86	Cytosol	0	0	190–708	PQ469197
	*hsp90b1*	800	91,965.43	4.73	Endoplasmic reticulum	0	21–22	261–782	PQ469198
	*trap1*	721	82,346.72	6.16	Mitochondrion	0	0	302–721	PQ469199

**Table 2:** Basic information of HSP70 and HSP90 gene families.

Gene Family	Gene Name	dN/dS	No. of Positive Sites	No. of Negative Sites
HSP70 family	*hspa1b*	0.0762	0	227
	*hspa4a*	0.136	0	120
	*hspa4b*	0.122	0	74
	*hspa4l*	0.136	0	250
	*hspa5*	0.121	0	165
	*hspa8*	0.0576	0	257
	*hspa9*	0.0995	0	240
	*hspa12a*	0.0755	0	169
	*hspa12b*	0.0764	0	232
	*hspa13*	0.107	0	172
	*hspa14*	0.165	0	149
HSP90 family	*hsp90aa1.1*	0.0583	0	158
	*hsp90aa1.2*	0.0462	0	217
	*hsp90ab1*	0.0575	0	212
	*hsp90b1*	0.0802	0	252
	*trap1*	0.100	0	233

## Data Availability

The original contributions presented in the study are included in the article/Supplementary Material, further inquiries can be directed to the corresponding author.

## Supplementary Materials

The following supporting information can be downloaded at https://www.mdpi.com/article/10.3390/genes15111445/s1, Table S1: Sequences of primers used in the present study; Table S2: Genes used in phylogenetic tree construction; Table S3: Genes used in selection pressure analysis; Table S4. Information on the chromosomal localization of HSPs.

## References

[1] Ren W., Ding B., Dong W., Yue Y., Long X., Zhou Z. (2024). Unveiling *HSP40/60/70/90/100* gene families and abiotic stress response in Jerusalem artichoke. Gene.

[2] Al-Whaibi M.H. (2011). Plant heat-shock proteins: A mini review. J. King Saud Univ.-Sci..

[3] Turan M. (2023). Genome-wide analysis and characterization of HSP gene families (HSP20, HSP40, HSP60, HSP70, HSP90) in the yellow fever mosquito (*Aedes aegypti*) (Diptera: Culicidae). J. Insect Sci..

[4] Sarkar N.K., Kim Y.-K., Grover A. (2009). Rice sHsp genes: Genomic organization and expression profiling under stress and development. BMC Genom..

[5] Deane E.E., Woo N.Y.S. (2011). Advances and perspectives on the regulation and expression of piscine heat shock proteins. Rev. Fish Biol. Fish..

[6] Zarei S., Ghafouri H., Vahdatiraad L., Heidari B. (2024). The influence of HSP inducers on salinity stress in sterlet sturgeon (*Acipenser ruthenus*): In vitro study on HSP expression, immune responses, and antioxidant capacity. Cell Stress Chaperones.

[7] He J., Wang J., Xu M., Wu C., Liu H. (2016). The cooperative expression of Heat Shock Protein 70 KD and 90 KD gene in juvenile *Larimichthys crocea* under *Vibrio alginolyticus* stress. Fish Shellfish Immunol..

[8] Paschoalini A.L., Ribeiro Y.M., Thuller B., Soares C.L.G., Rizzo E., Bazzoli N. (2024). Histopathology and changes in the expression of metallothioneins, heat shock proteins and inducible nitric oxide synthase in *Prochilodus costatus* from a neotropical river contaminated by heavy metals. Environ. Toxicol. Pharmacol..

[9] Wang X.-Q., Zheng N.-H., Cui C.-H., Cao M., Shen Y., Liu Y., Zhang Z.-W., Tang Z.-W., Qin C.-X., Chen B.-Y. (2021). Excavation of heat shock protein gene and its response to low salinity stress in *Sepia esculenta*. Mar. Sci..

[10] Lu H., Liu C., Yang C., He Z., Wang L., Song L. (2024). Genome-wide identification of the HSP70 genes in Pacific oyster *Magallana gigas* and their response to heat stress. Cell Stress Chaperones.

[11] Zhu Y., Dai Z. (2024). HSP90: A promising target for NSCLC treatments. Eur. J. Pharmacol..

[12] Ke Y., Xu M., Hwarari D., Chen J., Yang L. (2022). Genomic survey of heat shock proteins in *Liriodendron chinense* provides insight into evolution, characterization, and functional diversities. Int. J. Mol. Sci..

[13] Sun Y.-Y., Guo H.-Y., Liu B.-S., Zhang N., Zhu K.-C., Xian L., Zhao P.-H., Yang H.-Y., Zhang D.-C. (2024). Genome-wide identification of heat shock protein gene family and their responses to pathogen challenge in *Trachinotus ovatus*. Fish Shellfish Immunol..

[14] Islam M.J., Kunzmann A., Slater M.J. (2021). Extreme winter cold-induced osmoregulatory, metabolic, and physiological responses in European seabass (*Dicentrarchus labrax*) acclimatized at different salinities. Sci. Total Environ..

[15] Song Y., Han Z., Lu Z., Jiang Y., He Y., Tu K., Que H. (2024). Acquired thermotolerance in *Crassostrea angulata*: Effects of priming temperature, recovery temperature and duration on the survival and expression of HSP genes. Aquaculture.

[16] Sutili F.J., Gressler L.T., Vargas A.C., Zeppenfeld C.C., Baldisserotto B., Cunha M.A. (2013). The use of nitazoxanide against the pathogens *Ichthyophthirius multifiliis* and *Aeromonas hydrophila* in silver catfish (*Rhamdia quelen*). Vet. Parasitol..

[17] Gao J.-H., Zhao J.-L., Yao X.-L., Tola T., Zheng J., Xue W.-B., Wang D.-W., Xing Y. (2024). Identification of antimicrobial peptide genes from transcriptomes in Mandarin fish (*Siniperca chuatsi*) and their response to infection with *Aeromonas hydrophila*. Fish Shellfish Immunol..

[18] Jørgensen L.v.G. (2017). The fish parasite *Ichthyophthirius multifiliis*—Host immunology, vaccines and novel treatments. Fish Shellfish Immunol..

[19] Yunis-Aguinaga J., Sotil G., Morey G.A.M., Fernandez-Espinel C., Flores-Dominick V., Rengifo-Marin G., da Silva Claudiano G., Medina-Morillo M. (2024). Susceptibility of the cultured Amazonian fish, *Colossoma macropomum*, to experimental infection with *Aeromonas* species from ornamental fish. Microb. Pathog..

[20] Aly S., El-gheit E.-S.A., Osman H., Tolba M.M., Essameldin H.M., Fathi M. (2024). Cumulative assessment of *Diplectanum* spp. occurrence, prevalence, and pathological impact in *Dicentrarchus labrax* from varied Egyptian fish farms. Vet. Parasitol..

[21] Islam M.J., Kunzmann A., Slater M.J. (2022). Responses of aquaculture fish to climate change-induced extreme temperatures: A review. J. World Aquac. Soc..

[22] Boratyn G.M., Camacho C., Cooper P.S., Coulouris G., Fong A., Ma N., Madden T.L., Matten W.T., McGinnis S.D., Merezhuk Y. (2013). BLAST: A more efficient report with usability improvements. Nucleic Acids Res..

[23] Letunic I., Khedkar S., Bork P. (2021). SMART: Recent updates, new developments and status in 2020. Nucleic Acids Res..

[24] Ji J.X., Zhang L., Li L., Wang K.L., Hou J., Liu L.H., Li B., Zhang B.D., Li N., Chen S.N. (2023). Molecular cloning and functional analysis of polymeric immunoglobulin receptor, pIgR, gene in mandarin fish *Siniperca chuatsi*. Fish Shellfish Immunol..

[25] Smiline Girija A.S. (2020). Delineating the immuno-dominant antigenic vaccine peptides against gacS-Sensor kinase in *Acinetobacter baumannii*: An in silico investigational approach. Front. Microbiol..

[26] Chao J., Li Z., Sun Y., Aluko O.O., Wu X., Wang Q., Liu G. (2021). MG2C: A user-friendly online tool for drawing genetic maps. Mol. Hortic..

[27] Bailey T.L., Boden M., Buske F.A., Frith M., Grant C.E., Clementi L., Ren J., Li W.W., Noble W.S. (2009). MEME SUITE: Tools for motif discovery and searching. Nucleic Acids Res..

[28] Liu Y.-L., Zhou Y., Cao D., Ma L.-L., Gong Z.-M., Jin X.-F. (2020). Application analysis of predictors for plant protein subcellular localization based on proteome data of *Camellia sinensis* (L.) O. Ktze. Plant Sci. J..

[29] Qiao Y., Yan W., He J., Liu X., Zhang Q., Wang X. (2021). Identification, evolution and expression analyses of mapk gene family in Japanese flounder (*Paralichthys olivaceus*) provide insight into its divergent functions on biotic and abiotic stresses response. Aquat. Toxicol..

[30] Weaver S., Shank S.D., Spielman S.J., Li M., Muse S.V., Kosakovsky Pond S.L. (2018). Datamonkey 2.0: A modern web application for characterizing selective and other evolutionary processes. Mol. Biol. Evol..

[31] Zhang W., Shen M., Chu P., Wang T., Ji J., Ning X., Yin S., Zhang K. (2024). Molecular characterization of CIRBP from *Takifugu fasciatus* and its potential roles in cold-induced liver damage. Int. J. Biol. Macromol..

[32] Guo X., Qian Z., Pan Q., Hu Y., Mei W., Xing X., Yin S., Ji J., Zhang K. (2023). Effects of florfenicol on intestinal histology, apoptosis and gut microbiota of Chinese mitten crab (*Eriocheir sinensis*). Int. J. Mol. Sci..

[33] Livak K.J., Schmittgen T.D. (2001). Analysis of relative gene expression data using real-time quantitative PCR and the 2(-Delta Delta C(T)) Method. Methods.

[34] Kastano K., Mier P., Andrade-Navarro M.A. (2021). The role of low complexity regions in protein interaction modes: An illustration in huntingtin. Int. J. Mol. Sci..

[35] Zhang Y., Li J., Chu P., Shang R., Yin S., Wang T. (2023). Construction of a high-density genetic linkage map and QTL mapping of growth and cold tolerance traits in *Takifugu fasciatus*. BMC Genom..

[36] Suresh K., Pillai D. (2023). Prevalence and characterization of virulence-associated genes and antimicrobial resistance in *Aeromonas hydrophila* from freshwater finfish farms in Andhra Pradesh, India. Biologia.

[37] Araújo B.d.L., Serantoni Moyses C.R., Spadacci-Morena D.D., Xavier J.G., Lallo M.A. (2024). White spots amidst the gold: Ultrastructural and histological aspects of the chronic inflammatory response of goldfish with ichthyophthiriasis. J. Comp. Pathol..

[38] Long Y., Song G., Yan J., He X., Li Q., Cui Z. (2013). Transcriptomic characterization of cold acclimation in larval zebrafish. BMC Genom..

[39] Ryan J.A., Hightower L.E., Feige U., Yahara I., Morimoto R.I., Polla B.S. (1996). Stress proteins as molecular biomarkers for environmental toxicology. Stress-Inducible Cellular Responses.

[40] Cui Y., Liu B., Xie J., Xu P., Habte-Tsion H.M., Zhang Y. (2014). Effect of heat stress and recovery on viability, oxidative damage, and heat shock protein expression in hepatic cells of grass carp (*Ctenopharyngodon idellus*). Fish Physiol. Biochem..

[41] Dayrit G.B., Burigsay N.P.F., Vera Cruz E.M., Santos M.D. (2024). In silico characterization and homology modeling of Nile tilapia (*Oreochromis niloticus*) Hsp70cBi and Hsp70cBc proteins. Heliyon.

[42] Vollmer W., Shively J.M. (2006). Cytoskeletal elements in prokaryotes. Complex Intracellular Structures in Prokaryotes.

[43] Zhang T., Wang S., Jiang Y., Zhou H., Sun M., Ning Z., Mu W. (2023). Identification, characterization, and function of GRP94 and HSP90β in cold stress response in cold water fish *Phoxinus lagowskii*. Aquac. Rep..

[44] Zwonitzer K.D., Iverson E.N.K., Sterling J.E., Weaver R.J., Maclaine B.A., Havird J.C. (2023). Disentangling positive selection from relaxed selection in animal mitochondrial genomes. Am. Nat..

[45] Liu X., Wang H., Liang X., Roberts M.S., Muriel P. (2017). Chapter 30—Hepatic Metabolism in Liver Health and Disease. Liver Pathophysiology.

[46] Peng H., Yang B., Li B., Cai Z., Cui Q., Chen M., Liu X., Yang X., Jiang C. (2019). Comparative transcriptomic analysis reveals the gene expression profiles in the liver and spleen of Japanese pufferfish (*Takifugu rubripes*) in response to *Vibrio harveyi* infection. Fish Shellfish Immunol..

[47] Abarike E.D., Jian J., Tang J., Cai J., Sakyi E.M., Kuebutornye F.K.A. (2020). A mixture of Chinese herbs and a commercial probiotic *Bacillus* species improves hemato-immunological, stress, and antioxidant parameters, and expression of HSP70 and HIF-1α mRNA to hypoxia, cold, and heat stress in Nile tilapia, *Oreochromis niloticus*. Aquac. Rep..

[48] Peng G., Zhao W., Shi Z., Chen H., Liu Y., Wei J., Gao F. (2016). Cloning HSP70 and HSP90 genes of kaluga (*Huso dauricus*) and the effects of temperature and salinity stress on their gene expression. Cell Stress Chaperones.

[49] Annamaneni S., Vishwakarma S.K., Meka P.B., Khan A.A., Nallari P. (2019). Regulation of heat shock protein-70 gene transcripts in breast cancer cells during hypo and hyperthermia exposure. Meta Gene.

[50] Baharloei M., Heidari B., Zamani H., Ghafouri H., Hadavi M. (2021). Effects of heat shock protein inducer on Hsp70 gene expression and immune parameters during *Streptococcus iniae* infection in a Persian sturgeon fry. Vet. Res. Forum.

[51] Das S., Mohapatra A., Sahoo P.K. (2015). Expression analysis of heat shock protein genes during *Aeromonas hydrophila* infection in rohu, *Labeo rohita*, with special reference to molecular characterization of Grp78. Cell Stress Chaperones.

[52] Deane E.E., Li J., Woo N.Y.S. (2004). Modulated heat shock protein expression during pathogenic *Vibrio alginolyticus* stress of sea bream. Dis. Aquat. Org..

[53] Deane E.E., Woo N.Y.S. (2005). Evidence for disruption of Na^+^-K^+^-ATPase and hsp70 during vibriosis of sea bream, *Sparus* (*=Rhabdosargus*) *sarba* Forsskål. J. Fish Dis..

[54] Guo S., Wan Q., Xu M., Chen M., Chen Z. (2024). Transcriptome analysis of host *anti-Aeromonas hydrophila* infection revealed the pathogenicity of *A. hydrophila* to American eels (*Anguilla rostrata*). Fish Shellfish Immunol..

[55] Núñez-Díaz J.A., Fumanal M., Mancera J.M., Moriñigo M.A., Balebona M.C. (2016). Two routes of infection with *Photobacterium damselae* subsp. piscicida are effective in the modulation of the transcription of immune related genes in *Solea senegalensis*. Vet. Immunol. Immunopathol..

[56] Ibrahim D., Rahman M.M.I.A., El-Ghany A.M.A., Hassanen E.A.A., Al-Jabr O.A., El-Wahab R.A.A., Zayed S., Salem M.A.E.K., El_Tahawy S.N., Youssef W. (2024). *Chlorella vulgaris* extract conjugated magnetic iron nanoparticles in nile tilapia (*Oreochromis niloticus*): Growth promoting, immunostimulant and antioxidant role and combating against the synergistic infection with *Ichthyophthirius multifiliis* and *Aeromonas hydrophila*. Fish Shellfish Immunol..

[57] Roh H., Kim D.-H. (2022). Identification, classification and functional characterization of HSP70s in rainbow trout (*Oncorhynchus mykiss*) through multi-omics approaches. Fish Shellfish Immunol..

[58] Cheng J., Xun X., Kong Y., Wang S., Yang Z., Li Y., Kong D., Wang S., Zhang L., Hu X. (2016). Hsp70 gene expansions in the scallop *Patinopecten yessoensis* and their expression regulation after exposure to the toxic dinoflagellate *Alexandrium catenella*. Fish Shellfish Immunol..

[59] You L., Ning X., Liu F., Zhao J., Wang Q., Wu H. (2013). The response profiles of HSPA12A and TCTP from *Mytilus galloprovincialis* to pathogen and cadmium challenge. Fish Shellfish Immunol..

