# Supplementary material for: Characterization of HSP70 and HSP90 Gene Family in Takifugu fasciatus and Their Expression Profiles on Biotic and Abiotic Stresses Response
Source: Genes (Basel). 2024 Nov 8;15(11):1445. doi: 10.3390/genes15111445 (PMC11593546; doi:10.3390/genes15111445)
Supplement: Supplementary file 1 [file genes-15-01445-s001.zip › genes-3221121-supplementary.pdf]

Table S1. Sequences of primers used in the present study.

Primer name	Sequences (5'→3')	Usage
hspa1b-F	TCACCATCACCAACGATAAGG	qPCR
hspa1b-R	CAGCTTTGTATTTGTCGGCG	qPCR
Hspa4a-F	ACGAGCAGTACAATCACCTG	qPCR
Hspa4a-R	GATCTCCTGAACCTTGACCAC	qPCR
Hspa4b-F	CGGAGGCATTGAAACAGTTG	qPCR
Hspa4b-R	ACCTCTTGAAACCTTGGACAG	qPCR
hspa4l-F	GTGGACCAGGAAGGTCAAGG	qPCR
hspa4l-R	CACAACCGGCAGATCAATGC	qPCR
hspa5-F	TCACCATCACAAACGACCAG	qPCR
hspa5-R	GCGTCAATCCTCTCCTTCAG	qPCR
hspa8-F	ACAACACCCGTCCAAAGGTT	qPCR
hspa8-R	GCGCTGCGAATCATTGAAGT	qPCR
hspa9-F	CAAAGATGCTGGTCAGATTGC	qPCR
hspa9-R	ACAGAAATGTCGAACGTACCG	qPCR
hspa12a-F	CCCACTCAATATCAACCTCCC	qPCR
hspa12a-R	TTCATCGCATCTGGACTCATC	qPCR
hspa12b-F	GGCATGATAACTCTGGACCTG	qPCR
hspa12b-R	ACGGCTGTGACTTTGATCTC	qPCR
hspa13-F	ACCCACAGAACACCATTTACG	qPCR
hspa13-R	TGATTAGCGGAGACAAGGAAC	qPCR
hspa14-F	CGCTGTCTTCCGTTTCCATAG	qPCR
hspa14-R	TCCCTCTTCATGGTCAACAC	qPCR
hsp90aa1.1-F	CAGTTCGGAGTGGGTTTCTAC	qPCR
hsp90aa1.1-R	CGTCATCGTTGTGCTTTGTG	qPCR
hsp90aa1.2-F	TTTCTACTCCGCCTACCT	qPCR
hsp90aa1.2-R	TTTGACTCTTCGCTCCTC	qPCR
hsp90ab1-F	GAGTCTGACCAACGACTGGG	qPCR

hsp90ab1-R	GGGATGAGCTCTTCGCAGTT	qPCR
hsp90b1-F	AGAAGACGACCTGGGGAA	qPCR
hsp90b1-R	GTCAGAGACAGCAAACGG	qPCR
trap1-F	TTCTACTCCGCTTTCATGGTG	qPCR
trap1-R	ACGATCTTGGTTCCCTGTTG	qPCR

Table S2. Genes used in phylogenetic tree construction.

Gene	Species	NCBI reference sequence
hspa1b	*Takifugu rubripes*	XP_003963154.1
	Danio rerio	NP_001093532.1
	Mus musculus	NP_034608.2
	Homo sapiens	NP_005337.2
	Oncorhynchus mykiss	XP_036796849.1
	Esox lucius	XP_028980157.2
	Oryzias latipes	NP_001098384.1
	Scophthalmus maximus	XP_035500186.1
hspa4	*Mus musculus*	NP_0032326.3
	Homo sapiens	NP_002145.3
	Xenopus tropicalis	NP_989252.1
	Gallus gallus	XP_046783064.1
hspa4a	*Takifugu rubripes*	XP_011609590.1
	Danio rerio	NP_999881.1
	Oncorhynchus mykiss	XP_021473142.1
	Ictalurus punctatus	XP_017348513.1

	Oreochromis niloticus	XP_021473142.1
	Scophthalmus maximus	XP_035480912.1
hspa4b	*Danio rerio*	NP_956151.1
	Ictalurus punctatus	XP_017340400.1
	Takifugu rubripes	XP_011609027.2
hspa4l	*Mus musculus*	NP_035150.3
	Homo sapiens	NP_055093.2
	Gallus gallus	NP_001012594.2
	Danio rerio	XP_690505.2
	Ictalurus punctatus	XP_017330896.1
	Esox lucius	XP_010877156.1
	Oncorhynchus mykiss	XP_021429967.2
	Takifugu rubripes	XP_003962711.2
	Oreochromis niloticus	XP_003453147.1
	Scophthalmus maximus	XP_035465249.1
	Oryzias latipes	XP_004082341.1
hspa5	*Mus musculus*	NP_001126906.1
	Homo sapiens	NP_005338.1
	Gallus gallus	NP_990822.1
	Xenopus tropicalis	XP_002941690.1
	Ictalurus punctatus	XP_017315801.1
	Danio rerio	NP_998223.1
	Esox lucius	XP_010876087.1

	Oncorhynchus mykiss	XP_021458130.2
	Takifugu rubripes	XP_003965205.1
	Oreochromis niloticus	XP_025761207.1
	Scophthalmus maximus	XP_035464205.1
	Oryzias latipes	NP_001265730.1
hspa8	*Mus musculus*	NP_112442.2
	Homo sapiens	NP_006588.1
	Gallus gallus	NP_990334.2
	Xenopus tropicalis	XP_012823022.1
	Ictalurus punctatus	NP_001187202.1
	Danio rerio	NP_001103873.1
	Esox lucius	XP_019896696.1
	Oncorhynchus mykiss	XP_021480498.2
	Takifugu rubripes	XP_029703818.1
	Oreochromis niloticus	XP_019220380.1
	Scophthalmus maximus	XP_035480669.1
	Oryzias latipes	NP_001098270.1
hspa9	*Mus musculus*	NP_034611.2
	Homo sapiens	NP_004125.3
	Gallus gallus	NP_001006147.2
	Xenopus tropicalis	NP_001001229.1
	Ictalurus punctatus	XP_017330210.1
	Danio rerio	NP_958483.2

	Esox lucius	XP_010897601.1
	Oncorhynchus mykiss	XP_021416481.2
	Takifugu rubripes	XP_029703156.1
	Oreochromis niloticus	XP_003459471.1
	Scophthalmus maximus	XP_035504920.1
	Oryzias latipes	XP_023815147.1
hspa12a	*Mus musculus*	NP_001314927.1
	Homo sapiens	NP_001317093.1
	Gallus gallus	XP_040530742.1
	Xenopus tropicalis	XP_002941887.2
	Ictalurus punctatus	XP_017331518.1
	Danio rerio	XP_017207164.1
	Esox lucius	XP_010868569.1
	Oncorhynchus mykiss	XP_036839771.1
	Takifugu rubripes	XP_029687854.1
	Oreochromis niloticus	XP_025753215.1
	Scophthalmus maximus	XP_035461187.2
	Oryzias latipes	XP_004076967.1
hspa12b	*Mus musculus*	NP_082582.1
	Homo sapiens	NP_443202.3
	Xenopus tropicalis	XP_002936323.2
	Ictalurus punctatus	XP_017327892.1
	Danio rerio	NP_001036151.1

	Esox lucius	XP_012995772.1
	Oncorhynchus mykiss	XP_036843121.1
	Takifugu rubripes	XP_003977970.1
	Oreochromis niloticus	XP_025760390.1
	Scophthalmus maximus	XP_047184573.1
	Oryzias latipes	XP_004079822.1
hsp13	*Mus musculus*	NP_084477.1
	Homo sapiens	NP_008879.3
	Gallus gallus	NP_001025964.3
	Xenopus tropicalis	NP_001017223.1
	Ictalurus punctatus	XP_017345941.1
	Danio rerio	NP_001082948.1
	Esox lucius	XP_010875532.2
	Oncorhynchus mykiss	XP_021438575.2
	Takifugu rubripes	XP_003968291.1
	Oreochromis niloticus	XP_003441638.1
	Scophthalmus maximus	XP_035478781.2
	Oryzias latipes	XP_004075919.1
hsp14	*Mus musculus*	NP_056580.2
	Homo sapiens	NP_057383.2
	Gallus gallus	NP_001383360.1
	Xenopus tropicalis	NP_001015780.1
	Ictalurus punctatus	XP_017350154.1

	Danio rerio	NP_001038541.1
	Esox lucius	XP_010876776.2
	Oncorhynchus mykiss	XP_036828536.1
	Takifugu rubripes	XP_029681129.1
	Oreochromis niloticus	XP_003455685.1
	Scophthalmus maximus	XP_035479932.2
	Oryzias latipes	XP_004083036.1
hsp90aa1.1	*Mus musculus*	NP_034610.1
	Homo sapiens	NP_005339.3
	Gallus gallus	NP_001103255.2
	Xenopus tropicalis	NP_001016282.1
	Ictalurus punctatus	XP_017311367.1
	Danio rerio	NP_571403.1
	Esox lucius	XP_010902983.3
	Oncorhynchus mykiss	XP_021468115.2
	Takifugu rubripes	XP_003971591.1
	Oreochromis niloticus	XP_003440693.1
	Scophthalmus maximus	XP_035473086.1
	Oryzias latipes	XP_004083818.1
hsp90aa1.2	*Ictalurus punctatus*	XP_017311366.1
	Danio rerio	NP_001038538.1
	Esox lucius	XP_010902984.1
	Takifugu rubripes	XP_003971590.1

	Oreochromis niloticus	XP_003440692.1
	Scophthalmus maximus	XP_035473085.1
	Oryzias latipes	XP_004083819.1
hsp90ab1	*Mus musculus*	NP_032328.2
	Homo sapiens	NP_031381.2
	Gallus gallus	NP_001384246.1
	Xenopus tropicalis	NP_001025655.1
	Ictalurus punctatus	NP_001316242.1
	Danio rerio	NP_571385.2
	Esox lucius	XP_010876683.1
	Oncorhynchus mykiss	NP_001117703.1
	Takifugu rubripes	XP_003971791.1
	Oreochromis niloticus	XP_013127438.1
	Oryzias latipes	XP_020570462.1
hsp90b1	*Mus musculus*	NP_035761.1
	Homo sapiens	NP_003290.1
	Gallus gallus	NP_989620.22
	Xenopus tropicalis	NP_0010139228.1
	Ictalurus punctatus	XP_017349754.1
	Danio rerio	NP_937853.1
	Esox lucius	XP_010885352.2
	Oncorhynchus mykiss	XP_036813282.1
	Takifugu rubripes	XP_003967565.1

	Oreochromis niloticus	XP_003443932.1
	Oryzias latipes	XP_020559565.1
	Scophthalmus maximus	XP_035498793.2
trap1	*Mus musculus*	NP_080784.1
	Homo sapiens	NP_001258978.1
	Gallus gallus	NP_001006175.2
	Xenopus tropicalis	XP_002932506.1
	Ictalurus punctatus	XP_017308199.1
	Danio rerio	NP_001107097.1
	Esox lucius	XP_010887746.2
	Oncorhynchus mykiss	XP_036794987.1
	Takifugu rubripes	XP_003964711.1
	Oreochromis niloticus	XP_003450104.1
	Oryzias latipes	XP_004071480.1
	Scophthalmus maximus	XP_035470112.2

Table S3. Genes used in selection pressure analysis.

Species name	Genes	Accession No.
Takifugu rubripes	*hspa1b*	XP_003963154.1
	hspa4a	XP_011609590.1
	hspa4b	XP_011609027.2
	hspa4l	XP_003962711.2
	hspa5	XP_003965205.1
	hspa8	XP_029703818.1
	hspa9	XP_029703156.1
	hspa12a	XP_029687854.1
	hspa12b	XP_003977970.1

	hspa13	XP_003968291.1
	hspa14	XP_029681129.1
	hsp90aa1.1	XP_003971591.1
	hsp90aa1.2	XP_003971590.1
	hsp90ab1	XP_003971791.1
	hsp90b1	XP_003967565.1
	trap1	XP_003964711.1
Scophthalmus maximus	*hspa1b*	XP_035500186.1
	hspa4a	XP_035480912.1
	hspa4l	XP_035465249.1
	hspa5	XP_035464205.1
	hspa8	XP_035480669.1
	hspa9	XP_035504920.1
	hspa12a	XP_035461187.2
	hspa12b	XP_047184573.1
	hspa13	XP_035478781.2
	hspa14	XP_035479932.2
	hsp90aa1.1	XP_035473086.1
	hsp90aa1.2	XP_035473085.1
	hsp90ab1	XP_035473497.1
	hsp90b1	XP_035498793.2
	trap1	XP_035470112.2
Oreochromis niloticus	*hspa4l*	XP_003453147.1
	hspa5	XP_025761207.1
	hspa8	XP_019220380.1
	hspa9	XP_035504920.1
	hspa12a	XP_035461187.2
	hspa12b	XP_047184573.1
	hspa13	XP_003441638.1
	hspa14	XP_003455685.1
	hsp90aa1.1	XP_035473086.1
	hsp90aa1.2	XP_003440692.1
	hsp90ab1	XP_013127438.1
	hsp90b1	XP_003443932.1

	trap1	XP_003450104.1
Ictalurus punctatus	*hspa4a*	XP_017348513.1
	hspa4b	XP_017340400.1
	hspa4l	XP_017330896.1
	hspa5	XP_017315801.1
	hspa8	NP_001187202.1
	hspa9	XP_017330210.1
	hspa12a	XP_017331518.1
	hspa12b	XP_017327892.1
	hspa13	XP_017345941.1
	hspa14	XP_017350154.1
	hsp90aa1.1	XP_017311367.1
	hsp90aa1.2	XP_017311366.1
	hsp90ab1	NP_001316242.1
	hsp90b1	XP_017349754.1
	trap1	XP_017308199.1
Danio rerio	*hspa1b*	NP_001093532.1
	hspa4a	NP_999881.1
	hspa4b	NP_956151.1
	hspa4l	XP_690505.2
	hspa5	NP_998223.1
	hspa8	NP_001103873.1
	hspa9	NP_958483.2
	hspa12a	XP_017207164.1
	hspa12b	NP_001036151.1
	hspa13	NP_001082948.1
	hspa14	NP_001038541.1
	hsp90aa1.1	NP_571403.1
	hsp90aa1.2	NP_001038538.1
	hsp90ab1	NP_571385.2
	hsp90b1	NP_937853.1
	trap1	NP_001107097.1
Oncorhynchus mykiss	*hspa1b*	XP_036796849.1
	hspa4l	XP_021429967.2

	hspa5	XP_021458130.2
	hspa8	XP_021480498.2
	hspa9	XP_021416481.2
	hspa12a	XP_036839771.1
	hspa12b	XP_036843121.1
	hspa13	XP_021438575.2
	hspa14	XP_036828536.1
	hsp90aa1.1	XP_021468115.2
	hsp90ab1	NP_001117703.1
	hsp90b1	XP_036813282.1
	trap1	XP_036794987.1
Esox lucius	*hspa1b*	XP_028980157.2
	hspa4a	XP_010869421.1
	hspa4b	XP_010895421.1
	hspa4l	XP_010877156.1
	hspa5	XP_010876087.1
	hspa8	XP_019896696.1
	hspa9	XP_010897601.1
	hspa12a	XP_010868569.1
	hspa12b	XP_012995772.1
	hspa13	XP_010875532.2
	hspa14	XP_036828536.1
	hsp90aa1.1	XP_010902983.3
	hsp90aa1.2	XP_010902984.1
	hsp90ab1	XP_010876683.1
	hsp90b1	XP_010885352.2
	trap1	XP_010887746.2
Oryzias latipes	*hspa1b*	NP_001098384.1
	hspa4l	XP_004082341.1
	hspa5	NP_001265730.1
	hspa8	NP_001098270.1
	hspa9	XP_023815147.1
	hspa12a	XP_004076967.1
	hspa12b	XP_004079822.1

	hspa13	XP_004075919.1
	hspa14	XP_004083036.1
	hsp90aa1.1	XP_004083818.1
	hsp90aa1.2	XP_004083819.1
	hsp90ab1	XP_020570462.1
	hsp90b1	XP_020559565.1
	trap1	XP_004071480.1

Table S4. Information on the chromosomal localization of HSPs.

Genes	Chromosomal localization
hspa1b	Chr15: 1622030-1630969
hspa4a	Chr16: 7011863-7013018
hspa4b	Chr12: 9058935-9069712
hspa4l	Chr14: 6281083-6286212
hspa5	Chr20: 8784329-8786915
hspa8	Chr16: 433149-441493
hspa9	Chr12: 15697789-15726476
hspa12a	Chr6: 4262817-4289995
hspa12b	Chr4: 13119353-13127570
hspa13	Chr8: 13318324-13324076
hspa14	Chr13: 785601-789251
hsp90aa1.1	Chr18: 10443253-10445571
hsp90aa1.2	Chr18: 10445660-10448855
hsp90ab1	Chr18: 7087275-7109653
hsp90b1	Chr11: 13846504-13851132
trap1	Chr15: 6947148-6951213
